# NPDock: a web server for protein–nucleic acid docking

**DOI:** 10.1093/nar/gkv493

**Published:** 2015-05-14

**Authors:** Irina Tuszynska, Marcin Magnus, Katarzyna Jonak, Wayne Dawson, Janusz M. Bujnicki

**Affiliations:** 1 Laboratory of Bioinformatics and Protein Engineering, International Institute of Molecular and Cell Biology in Warsaw, ul. Ks. Trojdena 4, PL-02-109 Warsaw, Poland; 2 Institute of Informatics, University of Warsaw, Banacha 2, PL-02-097 Warsaw, Poland; 3 Bioinformatics Laboratory, Institute of Molecular Biology and Biotechnology, Faculty of Biology, Adam Mickiewicz University, ul. Umultowska 89, PL-61-614 Poznan, Poland

## Abstract

Protein–RNA and protein–DNA interactions play fundamental roles in many biological processes. A detailed understanding of these interactions requires knowledge about protein–nucleic acid complex structures. Because the experimental determination of these complexes is time-consuming and perhaps futile in some instances, we have focused on computational docking methods starting from the separate structures. Docking methods are widely employed to study protein–protein interactions; however, only a few methods have been made available to model protein–nucleic acid complexes. Here, we describe NPDock (Nucleic acid–Protein Docking); a novel web server for predicting complexes of protein–nucleic acid structures which implements a computational workflow that includes docking, scoring of poses, clustering of the best-scored models and refinement of the most promising solutions. The NPDock server provides a user-friendly interface and 3D visualization of the results. The smallest set of input data consists of a protein structure and a DNA or RNA structure in PDB format. Advanced options are available to control specific details of the docking process and obtain intermediate results. The web server is available at http://genesilico.pl/NPDock.

## INTRODUCTION

Proteins and nucleic acids are the two main types of biological macromolecules that often tend to function together in the cell. Protein–RNA and protein–DNA interactions play a fundamental role in a variety of biological processes, including DNA replication, RNA transcription, RNA splicing, degradation of nucleic acids and protein synthesis. These interactions are essential to cellular metabolism and the survival of all organisms. Defects in protein–nucleic acid interactions are implicated in a number of diseases, ranging from neurological disorders to cancer (1,2). Our understanding of these processes will improve as new structures of protein–nucleic acid complexes are solved and the structural details of the interactions are analyzed.

Typically, these details are obtained from crystallizing a given protein–RNA or protein–DNA complex. However, experimental determination of most protein–nucleic acid complex structures by high-resolution methods is a tedious and difficult process (3).

Computational techniques can complement experimental approaches in elucidating protein–nucleic acid interactions. In particular, docking methods aim at predicting the three-dimensional (3D) structures of macromolecular complexes, starting from the atomic coordinates of their components (4). Although less accurate than experimental measurements, theoretical models of macromolecular structures can yield sufficient information to build a working hypothesis and guide further experimental analyses to identify important amino acids or nucleotide residues.

The methodology for prediction and modeling of proteins and protein–protein complexes is very well established (5,6). Numerous protein–protein docking methods have been developed and assessed via the Critical Assessment of PRediction of Interactions (CAPRI) experiment (7). On the other hand, protein–nucleic acid docking, and specifically protein–RNA docking, has received relatively little attention from developers of computational methods. There are far fewer methods for predicting and modeling structures of nucleic acids other than strictly double-stranded DNA and RNA. In particular, methods for predicting the 3D structures of RNA molecules and protein–RNA complexes are relatively scarce (8,9). Programs for macromolecular docking that accept protein and RNA coordinates as an input include HADDOCK (10), GRAMM (11), HEX (12), PatchDock (13) and FTDock (14). These tools were originally developed for protein–protein docking and then adapted to accept nucleic acid molecules as receptors and/or ligands.

Most docking methods lack an intrinsic scoring function dedicated to assessing protein–RNA interactions. Such functions have been developed as standalone programs that allow for discriminating between well-docked and poorly docked complexes. For example, our group has developed statistical potentials QUASI-RNP and DARS-RNP that are deliberately coarse-grained to help take into account moderate conformational changes that may occur upon binding (15). Other methods for scoring protein–RNA complexes were also developed that take as an input pre-calculated complex structures and return scores (16–18). The combination of different programs for docking, scoring and selection of complex structures is relatively challenging for a typical user, in particular a biologist without programming skills. To facilitate protein–nucleic acid docking analyses, we developed a computational workflow that reads the input coordinates of a protein molecule and a nucleic acid molecule (RNA or DNA) and runs a series of established methods in a stepwise manner. The setup attempts to expedite file preparation by the user and the results are presented interactively, facilitating interpretation.

## MATERIALS AND METHODS

### Workflow implemented as NPDdock

**Figure 1.**
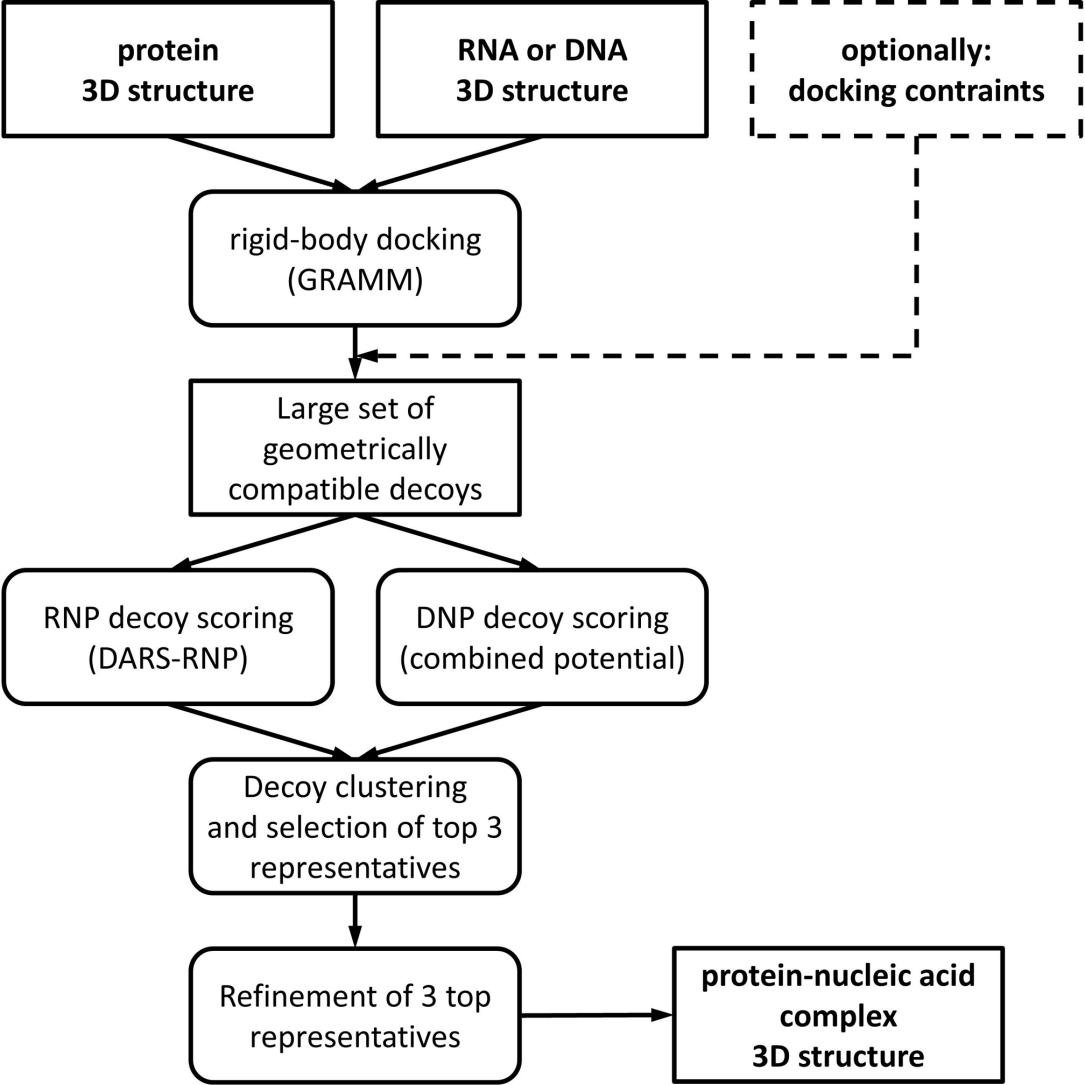
The workflow of the NPDock server.

NPDock is implemented as a computational workflow that consists of the GRAMM program, which is a third-party method (19), and a set of tools developed mainly in our laboratory, including the DARS-RNP and QUASI-RNP statistical potentials for scoring protein–RNA complexes with coarse-grained representation (15), a counterpart of QUASI-RNP for scoring protein–DNA complexes (QUASI-DNP), and tools for clustering, selection and refinement of models (Figure 1). For protein–DNA docking, we combined our in-house statistical potential with the DFIRE potential (20) and with a potential developed by the Varani group (21).

First, the GRAMM program is used to perform a rigid body global search and generate geometrically plausible protein–nucleic acid complex structures (decoys). Further analysis of decoys can be limited to those models that satisfy user-defined restraints; e.g., distance restraints between any set of residues defined by user. The decoys are scored and ranked using statistical potentials, developed for protein–RNA or protein–DNA complexes, respectively (see above). The best-scored decoys are then clustered using a procedure reported previously (22) and representatives of the three largest clusters are selected. Finally, a Monte Carlo Simulated Annealing (23) procedure (with protein and nucleic acid molecules treated as rigid bodies) is used to optimize the protein–nucleic acid interactions in these three cluster representatives, and the resulting structures of complexes are presented to the user.

### Required input files

The smallest set of input data consists of a protein structure file and a nucleic acid (DNA or RNA) structure file in PDB (both new and old), AMBER, CHARMM or mol2 formats. Mol2-formatted files are converted to the PDB format using OpenBabel (24). AMBER- and CHARMM-formatted files are converted via our parser written in Python. For even more robust conversion of formats, we recommend the use of third-party software; e.g., VMD (25). Following submission of the input files, the NPDock server checks the size of both molecules. Because of vector size limitations in the GRAMM program, the server can only process files up to 10 000 atoms each, and for larger files, it aborts the prediction and reports a problem. In such a case, we suggest resubmitting a docking job limited to parts of the input molecules that are most likely to interact with each other. Input structures must be formatted properly to be accepted by the GRAMM program. The most important conditions are described on the NPDock help page (http://iimcb.genesilico.pl/NPDock/help). Any atom that should be taken into consideration during the docking process, including non-standard residues and ligands, must be relabeled as ‘ATOM’: atoms labeled ‘HETATM’ in the input files will be ignored and may appear as ‘holes’ in the output structures. Improperly named atoms will not interfere with the docking process and will appear in the output; however, they will be ignored while evaluating the interactions and, hence, probably lead to an improper score.

### Parameters of docking

In addition to the main input files, the user is able to modify parameters used in the docking process. In particular, the input can include a list of interface residues for both the receptor and the ligand, and the number of protein–nucleic acid residue pairs (from the above-mentioned list) that are required to be in contact; i.e. at a distance ≤10 Å from each other. If the interface residues are provided for one molecule only, then all residues from the other molecule are permitted to form contacts. In general, restricting the docking procedure to a well-defined interface is likely to improve the accuracy of the docking prediction.

The user can also modify the parameters in the clustering procedure. By default, the 100 best-scored models are used for clustering. If no large clusters are identified, we suggest experimenting with a higher number of models (up to 1000). The default value for the RMSD threshold in the clustering procedure is set to 5 Å, which can be modified (typically increased) for structures that are very large or generate a large number of different poses. In our experience, values between 5 and 10 Å led to the most reasonable results.

The parameters of the rigid body refinement procedure can be also adjusted by the user. The number of simulation steps defines the length of the simulation. Long simulations allow the molecules to move out from a local minimum and may allow a more extensive sampling of the conformational landscape. However, long simulations are computationally costly; therefore, based on our experience, we recommend using values between 1000 and 10 000. The temperature of the first and last steps of the simulation defines the behavior of the Simulated Annealing algorithm. A high temperature allows for a larger freedom of movement; however, it should be combined with a longer simulation time to allow the system to cool down smoothly. We do not recommend changing the value of the last simulation step unless the user intends to perform very unorthodox sampling.

### Testing data sets

For protein–RNA docking, we used DARS-RNP and QUARI-RNP potentials that were trained earlier (15). For protein–DNA docking, we used a combination of three independent potentials: the DFIRE potential (20), the Varani potential (21) and a QUASI-DNP potential developed in the same manner as the QUASI-RNP potential, on a data set of protein–DNA complexes unrelated to those in the testing data set.

For testing of protein–RNA docking, we used the same 12 protein–RNA complexes from the Varani and Fernandez benchmarks used in our earlier work on the DARS-RNP and QUARI-RNP potentials (15) to compare the results of the NPDock server with those we obtained by manually using the implemented pipeline. Additionally, we tested NPDock on a much larger testing set of protein–RNA complexes (26). For testing of protein–DNA docking, we used a benchmark developed by van Dijk and Bonvin (27). Details of the data sets used for testing are described in the Supplementary data.

## RESULTS

### NPDock server

The NPDock server is designed to automate the procedure of protein–nucleic acid complex structure modeling. Published examples of structural models developed with the computational pipeline corresponding to NPDock include Trm14 methyltransferase complexed with tRNA (28), and an engineered RNaseH-zinc finger fusion protein complexed to an RNA–DNA hybrid (29). The server was launched in December 2013 as RNPdock and had the capability to dock protein–RNA complexes with the use of the DARS-RNP and QUASI-RNP potentials (15). In November 2014, the server was updated with a ‘meta-potential’ for scoring protein–DNA interactions, comprising three primary potentials: (i) a ‘protein–DNA’ version of the QUASI-RNP potential (QUASI-DNP, details to be published elsewhere), (ii) the DFIRE potential (20) and (iii) the Varani potential (21).

### Performance of the NPDock server for protein–RNA complexes

We tested the NPDock server on 13 protein–RNA complexes used in our original work on RNP potentials (15) (Supplementary Table S2). The results obtained here, by a fully automated procedure, were very similar to the results obtained previously employing a strategy of several time-consuming, independent manual methods by an expert user.

We have also tested the NPDock server on one of the available data sets for benchmarking protein–RNA computational docking methods (26). In the bound docking set, NPDock found near to native structures for 25/49 easy targets, 5/16 medium targets and 3/7 difficult targets (for jobs that were run without any information about the interaction site). When only one pair of interface residues was defined, NPDock recognized 33/49, 7/16 and 4/7 for easy, medium and difficult targets, respectively. For the unbound docking set, our server (run without information about the interaction site), recognized near to native structures for 19/49, 2/16 and 0/7 easy/medium/hard targets. When a single randomly selected interaction site pair was defined, the server found near to native structures for 29/49, 3/16 and 0/7 easy/medium/hard targets (Supplementary Table S3). These results clearly demonstrate that protein–RNA docking is very challenging and that the current docking methodology is generally able to generate reasonable predictions for complexes in which RNA and protein components do not change conformation upon binding, but cannot predict native-like structures if medium or large conformational changes occur. Further information can also be found in the Supplementary results.

### Performance of the NPDock server for protein–DNA complexes

For protein–DNA docking, we assessed the use of a combined ‘meta potential’ (K. Jonak, I. Tuszyńska, J.M. Bujnicki, details to be published elsewhere) that comprises QUASI-DNP, the DFIRE potential (20) and a potential developed by Varani *et al*. (21), on an independent testing set (27).

The results of testing the server obtained by the fully automated procedure were similar to the results obtained by the manual use of independent docking software and methods for scoring protein–DNA interactions (Supplementary Table S1). The benchmark is divided into easy, intermediate and difficult targets for docking. Therefore, we also analyzed the results by splitting the complexes into these three groups. For easy targets tested without restraints, 10/13 cases and 13/13 cases resulted in finding native-like structures for bound and unbound docking, respectively. For cases tested with a single defined residue restraint, 13/13 cases for the bound and 7/13 for the unbound found native-like structures. For intermediate targets, we obtained native-like decoys for 15/22 for the bound and 2/22 for the unbound cases. For intermediate targets with defined residues, we obtained 18/22 for the bound and 6/22 for unbound cases. For difficult targets, NPDock found 12/12 cases for bound docking with native-like structures and 3/12 for unbound docking without restraints and 4/12 with restraints. These results demonstrate that protein–DNA docking is also challenging, but generally easier than for protein–RNA docking, perhaps because of the more limited conformational changes in typical DNA structures. Further details can be found in the Supplementary results.

### Example uses

**Figure 2.**
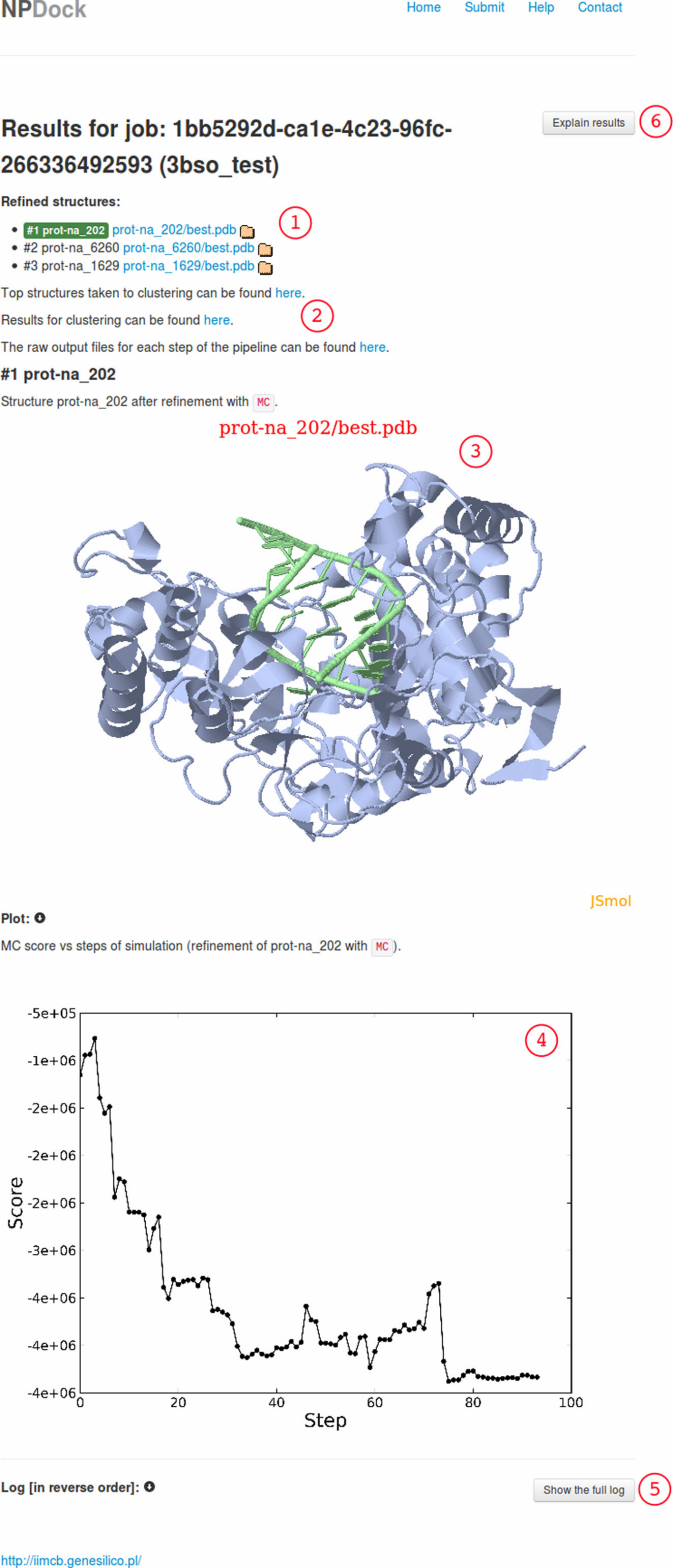
The NPDock result of docking of an apo-form of the Norwalk virus polymerase structure (PDB code: 1SH0) and dsRNA taken from that complex (PDB code: 3BSO): (1) a list of the refined best scored complexes, (2) raw files for each step of the docking can be viewed by clicking the links, (3) JSmol 3D visualization of the best scored complex, (4) a plot illustrating how the score changes during a simulation, (5) detailed information about each step of the docking can be viewed by clicking the ‘Show the full log’ button and (6) a detailed explanation of the result that can be viewed by clicking the ‘Explain results’ button.

A complete docking result consists of decoys and outputs from the docking process (Figure 2). At the top of the server results website, there are three PDB structures that are taken from the three largest clusters, refined and available for download, where the refined structure of the largest cluster is shown on the results website. A defined number of best scored structures that went into the clustering procedure are also available. Analysis of those structures can help the user to decide how to reuse the docking process with restraints to increase the number of correct structures that are clustered. Furthermore, the clustering results are available, so the user can download representatives from the other clusters if more than three clusters exist. The raw output files from each step of the docking pipeline are also available and the user can analyze the whole docking process and carry out additional data analysis if desired.

## DISCUSSION

NPDock is a novel web server developed for protein–nucleic acid docking that uses specific protein–nucleic acid statistical potentials for scoring and selection of modeled complexes. NPDock implements a unique workflow based on a combination of computational methods that have been published and offers a user-friendly web interface to enter PDB structures and view the results. The automation of the entire process makes the protein–nucleic acid docking available to users who would otherwise become tripped up installing many complex programs locally and then carrying out many manual steps; each requiring a variety of manual format conversions that are highly prone to human error. Therefore, it can help users save even more than ten times the time required to run different methods separately and sequentially.

Future plans are as follows. First, add additional potentials for protein–nucleic acid interactions (in particular for protein–RNA interactions), including potentials developed by third parties, as well as ones under development in our group. This extension may also encompass ‘meta’ scoring. Second, enable the docking of hybrid DNA–RNA molecules (e.g., dsRNA/DNA duplexes): we plan to implement a hybrid potential for simultaneous scoring of protein–RNA and protein–DNA interactions. Third, include a procedure of local flexible refinement of both protein and nucleic acid components, using methods such as REFINER (30) and SimRNA (31). These improvements may open the way to perform analyses of protein–nucleic acid complexes that undergo conformational changes upon interaction.

## AVAILABILITY

The web server is available at http://genesilico.pl/NPDock.

This website is free and open to all users and there is no login requirement.

## SUPPLEMENTARY DATA

Supplementary Data are available at NAR Online.

SUPPLEMENTARY DATA

## References

[1] Lukong K.E., Chang K.W., Khandjian E.W., Richard S. (2008). RNA-binding proteins in human genetic disease. Trends Genet..

[2] Cooper T.A., Wan L., Dreyfuss G. (2009). RNA and disease. Cell.

[3] Doudna J.A. (2000). Structural genomics of RNA. Nat. Struct. Biol..

[4] Rodrigues J.P., Bonvin A.M. (2014). Integrative computational modeling of protein interactions. FEBS J..

[5] Wichadakul D., McDermott J., Samudrala R. (2009). Prediction and integration of regulatory and protein-protein interactions. Methods Mol. Biol..

[6] Moreira I.S., Fernandes P.A., Ramos M.J. (2010). Protein-protein docking dealing with the unknown. J. Comp. Chem..

[7] Janin J. (2010). Protein-protein docking tested in blind predictions: the CAPRI experiment. Mol. Biosyst..

[8] Laing C., Schlick T. (2010). Computational approaches to 3D modeling of RNA. J. Phys. Condens Matter.

[9] Rother K., Rother M., Boniecki M., Puton T., Bujnicki J.M. (2011). RNA and protein 3D structure modeling: similarities and differences. J. Mol. Model..

[10] Dominguez C., Boelens R., Bonvin A.M. (2003). HADDOCK: a protein-protein docking approach based on biochemical or biophysical information. J. Am. Chem. Soc..

[11] Katchalski-Katzir E., Shariv I., Eisenstein M., Friesem A.A., Aflalo C., Vakser I.A. (1992). Molecular surface recognition: determination of geometric fit between proteins and their ligands by correlation techniques. Proc. Natl. Acad. Sci. U.S.A..

[12] Ritchie D.W., Kemp G.J. (2000). Protein docking using spherical polar Fourier correlations. Proteins.

[13] Schneidman-Duhovny D., Inbar Y., Nussinov R., Wolfson H.J. (2005). PatchDock and SymmDock: servers for rigid and symmetric docking. Nucleic Acids Res..

[14] Gabb H.A., Jackson R.M., Sternberg M.J. (1997). Modelling protein docking using shape complementarity, electrostatics and biochemical information. J. Mol. Biol..

[15] Tuszynska I., Bujnicki J.M. (2011). DARS-RNP and QUASI-RNP: new statistical potentials for protein-RNA docking. BMC Bioinformatics.

[16] Zheng S., Robertson T.A., Varani G. (2007). A knowledge-based potential function predicts the specificity and relative binding energy of RNA-binding proteins. FEBS J..

[17] Perez-Cano L., Solernou A., Pons C., Fernandez-Recio J. (2010). Structural prediction of protein-RNA interaction by computational docking with propensity-based statistical potentials. Pac. Symp. Biocomput..

[18] Huang S.Y., Zou X. (2014). A knowledge-based scoring function for protein-RNA interactions derived from a statistical mechanics-based iterative method. Nucleic Acids Res..

[19] Vakser I.A., Aflalo C. (1994). Hydrophobic docking: a proposed enhancement to molecular recognition techniques. Proteins.

[20] Zhang C., Liu S., Zhu Q., Zhou Y. (2005). A knowledge-based energy function for protein-ligand, protein-protein, and protein-DNA complexes. J. Med. Chem..

[21] Robertson T.A., Varani G. (2007). An all-atom, distance-dependent scoring function for the prediction of protein-DNA interactions from structure. Proteins.

[22] Shortle D., Simons K.T., Baker D. (1998). Clustering of low-energy conformations near the native structures of small proteins. Proc. Natl. Acad. Sci. U.S.A..

[23] Kirkpatrick S., Gelatt C.D. Jr, Vecchi M.P. (1983). Optimization by simulated annealing. Science.

[24] O'Boyle N.M., Banck M., James C.A., Morley C., Vandermeersch T., Hutchison G.R. (2011). Open Babel: an open chemical toolbox. J. Cheminform..

[25] Humphrey W., Dalke A., Schulten K. (1996). VMD: visual molecular dynamics. J. Mol. Graph..

[26] Huang S.Y., Zou X. (2013). A nonredundant structure dataset for benchmarking protein-RNA computational docking. J. Comput. Chem..

[27] van Dijk M., Bonvin A.M. (2008). A protein-DNA docking benchmark. Nucleic. Acids. Res..

[28] Fislage M., Roovers M., Tuszynska I., Bujnicki J.M., Droogmans L., Versees W. (2012). Crystal structures of the tRNA:m2G6 methyltransferase Trm14/TrmN from two domains of life. Nucleic Acids Res..

[29] Sulej A.A., Tuszynska I., Skowronek K.J., Nowotny M., Bujnicki J.M. (2012). Sequence-specific cleavage of the RNA strand in DNA-RNA hybrids by the fusion of ribonuclease H with a zinc finger. Nucleic Acids Res..

[30] Boniecki M., Rotkiewicz P., Skolnick J., Kolinski A. (2003). Protein fragment reconstruction using various modeling techniques. J. Comput. Aided Mol. Des..

[31] Rother K., Rother M., Boniecki M., Puton T., Tomala K., Lukasz P., Bujnicki J.M., Leontis NB, Westhof E (2012). Template-Based and Template-Free Modeling of RNA 3D Structure: Inspirations from Protein Structure Modeling. RNA 3D Structure Analysis and Prediction.

