# Supplementary material for: NPDock: a web server for protein–nucleic acid docking
Source: Nucleic Acids Res. 2015 May 14;43(Web Server issue):W425–30. doi: 10.1093/nar/gkv493 (PMC4489298; doi:10.1093/nar/gkv493)
Supplement: SUPPLEMENTARY DATA [file supp_43_W1_W425__index.html]

NPDock: a web server for protein–nucleic acid docking — SUPPLEMENTARY DATA 

# NPDock: a web server for protein–nucleic acid docking

## SUPPLEMENTARY DATA

- SUPPLEMENTARY DATA
