# Supplementary material for: NPDock: a web server for protein–nucleic acid docking
Source: Nucleic Acids Res. 2015 May 14;43(Web Server issue):W425–30. doi: 10.1093/nar/gkv493 (PMC4489298; doi:10.1093/nar/gkv493)
Supplement: SUPPLEMENTARY DATA [file supp_gkv493_nar-00680-web-b-2015-File004.docx]

**NPDock – a web server for protein-nucleic acid docking:**

**Supplementary data**

**Irina Tuszynska^1^, Marcin Magnus^1^, Katarzyna Jonak^1^, Wayne Dawson^1^, Janusz M. Bujnicki^1,2,*^**

^1^Laboratory of Bioinformatics and Protein Engineering, International Institute of Molecular and Cell Biology in Warsaw, ul. Ks. Trojdena 4, PL-02-109 Warsaw, Poland

^2^Bioinformatics Laboratory, Institute of Molecular Biology and Biotechnology, Faculty of Biology, Adam Mickiewicz University, ul. Umultowska 89, PL-61-614 Poznan, Poland

*Testing sets for protein-DNA docking:*

For testing of protein-DNA docking, we used a benchmark developed by van Dijk and Bonvin ([van Dijk and Bonvin, 2008](#_ENREF_4)). For manual testing of the “meta potential”, only the complexes with at least ten native-like structures generated by GRAMM docking were used: 34 for bound docking and 27 for unbound docking. The server was tested on all of the complexes from the benchmark: for 47 complexes, both bound and unbound docking experiments were performed. Due to the fact that many proteins consist of multiple subunits, 88 docking experiments were run considering the orientation of DNA with respect to various chains of the proteins in the native form of the complex. The benchmark is divided into three categories associated with the value of interface RMSD between the bound and unbound forms of the complexes: easy with 13/47 complexes (25/88 cases), intermediate with 22/47 complexes (41/88 cases) and difficult with 12/47 complexes (22/88 cases), where the () indicates docking experiments with the multiple subunits (typically dimers). In two complexes (1K79 and 1PT3), where the bound form of the complex consists of a single chain yet the unbound form is a dimer, two cases were considered during the docking procedure: first with a one chain and second with two chains of the protein taken for docking. The components for bound docking were obtained from the native structures of the complexes from the Protein Data Bank (<http://www.rcsb.org/>, PDB). For unbound docking, the proteins were obtained from the PDB, while B-DNA forms were generated using the model.it® server (<http://hydra.icgeb.trieste.it/dna/model_it.html>) ([Munteanu, et al., 1998](#_ENREF_2)).

*Testing sets for protein-RNA docking:*

For testing of protein-RNA docking, we used the same twelve protein-RNA complexes from the Varani and Fernandez benchmarks used in our earlier work on the DARS-RNP and QUARI-RNP potentials ([Tuszynska and Bujnicki, 2011](#_ENREF_3)) to compare the results of NPDock server with those we obtained by manually using of implemented pipeline. This dataset presented five exercises of bound docking (components taken from the complex) and seven exercises of unbound docking (where at least one of the components was solved experimentally in isolation from the complex). We also tested NPDock on a much larger testing set, published by Huang and Zou (<http://zoulab.dalton.missouri.edu/RNAbenchmark/>). This testing set consists of 72 targets for which both bound and unbound docking procedures were run. The testing set was divided into three categories (easy (49/72), medium (16/72) and difficult (7/72)) according to the degree of structural change in the individual components in the process of complex formation.

*Results of NPDock testing: protein-DNA complexes*

The results of testing the server obtained by a fully automated procedure were similar to the results obtained by manual use of independent docking software and methods for scoring protein-DNA interactions (Supplementary Table 1). From 34 complexes tested manually for bound docking, the server was able to recognize all of the native-like decoys recognized previously by a manually run pipeline – 29 in total. Additionally, the server recognized native-like decoys for three more complexes not recognized by the manual procedure: 1BDT, 1H9T and 1PT3 (tested as a single chain). Moreover, for a randomly chosen pair of interface residues, the server recognized three other complexes: 1DIZ, 1R4O and 1JTO. It is important to note that here, as a recognized complex, we consider that the native-like decoys were recognized for at least one orientation of DNA with respect to various chains of the proteins.

For unbound docking of 27 complexes, both manual testing and automatic using NPDock recognized eight native-like structures (for NPDock with or without defined pair of residues). The server did not recognize three of the complexes that were recognized by the manual testing: 1QRV, 1VAS and 1W0T. However, for 1QRV and 1VAS, NPDock recognized the binding site on the proteins. The server recognized seven complexes that were not recognized manually: 1DDN, 1G9Z, 1RPE, 2IRF and 4KTQ without restraints, as well as 1H9T and 1R4O, each with a single randomly defined interfacial residue. Additionally, NPDock found binding sites on the proteins 1BY4, 1CMA, 1DIZ and 1JT0.

For bound docking, in 54/88 tested cases of DNA orientations with respect to various chains of the proteins, the native-like structure was often found (38/47 when considering only one orientation for each complex). For the remaining cases (34/88), in 14 cases, the server found the binding sites on the proteins. Unrecognized complexes were tested with a defined single pair of interface residues. For testing with defined residues, we took one case from each unrecognized complex and two additional complexes that were recognized previously: 1PT3 and 1ZS4. For all of the 13 test complexes, the results improved, with 7/13 cases recognized. For the 6 unrecognized cases, in 4 the binding site on the protein was found.

For unbound docking, in 12/88 tested cases of DNA orientations with respect to various chains of the proteins, the native-like structure was found (8/47 when considering only one orientation for each complex). For unrecognized cases (76/88), in 23 cases, the server found the binding sites on the proteins. For testing with defined residues, we took one case from each unrecognized complex and four additional complexes that were recognized previously: 1PT3, 1KSY, 1QNE and 2IRF. For these four complexes, the results improved and again the native-like structures were recognized. For the remaining tested complexes, the server found native-like decoys for 9 cases. From unrecognized complexes (35), for 16 the binding site of the protein was found.

For the protein part of 1K79 and 1PT3, where the respective bound protein is a single subunit (monomer) but the unbound protein forms a dimer, we investigated if docking of the protein as a monomer or dimer would show a difference in the recognition of native-like structures. However, for both cases, we observed similar results. In bound docking, the native-like structures were recognized when docking the monomer or dimer. For unbound docking of 1K79, the binding site and the native-like structures where not found, while for 1PT3, the server again found native-like structure.

The benchmarks are divided into easy, intermediate and difficult targets for docking. Hence, we also analyzed the results by splitting the complexes into these three groups. For easy targets tested without restraints 10/13 cases and 13/13 cases resulted in finding native-like structures for bound and unbound docking, respectively. For cases tested with a single defined residue restraint, 13/13 cases for the bound and 7/13 for the unbound found native-like structures. For intermediate targets, we obtained native-like decoys for 15/22 for the bound and 2/22 for the unbound cases and for defined residues, 18/22 for the bound and 6/22 for unbound cases. For difficult targets the NPDock found 12/12 cases for bound docking with native-like structures and 3/12 for unbound docking without restraints and 4/12 with restraints.

The reason for many complexes with unrecognized native-like structures can be due to a failure in generating native-like decoys by GRAMM, like for 2FIO and 1O3T in unbound docking. However, there are also the cases where GRAMM generated near-native models, but NPDock did not recognize the native-like structures, for example 1BY4 and 2C5R (unbound set).

*Results of NPDock testing: protein-RNA complexes:*

We tested the server on thirteen protein-RNA complexes used in our original work on RNP potentials ([Tuszynska and Bujnicki, 2011](#_ENREF_3)) (Supplementary Table 2). The results obtained here, by a fully automated procedure, were very similar to the results obtained previously by time-consuming manual use of several independent methods by an expert user. In the bound docking test set (Varani and coworkers), both the server and the manually run pipeline recognized near to native decoys for four out of the five complexes tested. In the unbound testset (Fernandez and coworkers), NPDock reported native-like structures for three out of eight structures; it did not report a native-like structure for one complex that was successfully docked manually (2JEA). For this complex, the automated procedure implemented in the server placed the native-like structures within the fourth cluster, while the output considered for evaluation is limited to the three largest clusters only. However, if just one (randomly chosen) pair of interface residues is provided as a docking constraint, the server recognizes native-like structures for four out of the seven complexes that were unsuccessful in the previous step (docking without information about the interface).

We have also tested the NPDock server on one of the available datasets for benchmarking protein-RNA computational docking methods ([Huang and Zou, 2012](#_ENREF_1)). In a similar way as with the protein-DNA set, the benchmarks are divided into easy, intermediate and difficult targets for docking. In the bound docking set for easy, medium and difficult targets, NPDock found near to native structures for 25/49, 5/16 and 3/7 respectively (for jobs that were run without any information about the interaction site). When only one pair of interface residues was defined, NPDock recognized 33/49, 7/16 and 4/7 for easy, medium and difficult tragets. For the unbound docking set, our server (run without information about the interaction site), recognized near to native structures for 19/49, 2/16 and 0/7 complexes while, when a randomly selected interaction site pair was defined, the server found near to native structures for 29/49, 3/16 and 0/7 targets. Besides this, there are complexes (1 for bound docking and 3 for unbound docking) for which near to native structures were recognized, but with an RMSD > 10 Å, as well as those for which the native site of either protein or RNA was recognized (7 for bound docking and 10 for unbound docking).

**Supplementary Table 1.** Results of fully automated protein-DNA docking with the NPDock server; comparison with user-supervised docking.

| **BOUND DOCKING** | | | | | |
| --- | --- | --- | --- | --- | --- |
| **Complex name** | **Complex PDB code** | **Protein chain name (docked chain in respect to native chain)** | **RMSD of ligand [Å] unrefined**  **(manual testing - approximately)** | **RMSD of ligand [Å] refined**  **(free docking without interface residues pair)** | **RMSD of ligand [Å] refined**  **(docking with a constraint on one random pair on interface residues)** |
| ‘Easy’ targets | | | | | |
| Phage PHI29 replication organizer protein P16.7 | 2c5r | A-B/A-B | - | 37.47 | 13.86* |
|  |  | A-B/B-A | - | 37.61 | - |
|  |  | A-B/C-D | - | 37.21 | 7.67 |
|  |  | A-B/D-C | - | 35.53 | - |
|  |  | A-B/E-F | - | 38.05 | 13.29* |
|  |  | A-B/F-E | - | 34.27 | - |
| Col-E7 nuclease domain | 1pt3^ | A/A | 36.50 | 7.56 | - |
|  |  | A-B/A-B | - | 13.09^#^ | 5.95 |
|  |  | A-B/B-A | - | 12.11^#^ | - |
| Sporulation specific transcription factor NDT80 | 1mnn^ | A/A | 3.80 | 2.79 | - |
| Restriction endonuclease FOKI | 1fok^ | A/A | 3.40 | 0.33 | - |
| Papillomavirus replication initiation domain E-1 | 1ksy | A-C/A-C | - | 29.37 | 9.20 |
|  |  | A-C/C-A | - | 45.89 | - |
| Phage 434 CRO | 3cro^ | L/L | 1.20 | 5.44 | - |
|  |  | L/R | 15.60^#^ | 39.21* | - |
| Human uracil-DNA glucosylase | 1emh^ | A/A | 10.80^#^ | 7.65 | - |
| FADR, fatty acid responsive transcription factor | 1h9t^ | A-B/A-B | 35.50 | 1.59 | - |
|  |  | A-B/B-A | - | 3.14 | - |
| TRP repressor | 1tro | A-C/A-C | - | 1.12 | - |
|  |  | A-C/C-A | - | 23.37 | - |
| Retinoid X receptor DNA binding domain | 1by4^ | A/A | 4.50 | 26.12* | - |
|  |  | A/B | - | 1.70 | - |
| RUNX1 runt domain | 1hjc | A/A | - | 8.17 | - |
| *E. coli* 3-methyladenine DNA glycosylase II | 1diz^ | A/A | no decoys clustered | 43.78 | 1.05 |
| Phage 434 repressor | 1rpe^ | L/L | 11.80^#^ | 38.79* | - |
|  |  | L/R | 1.20 | 4.03 | - |
| ‘Medium’ targets | | | | | |
| Restriction endonuclease BSTYI | 1vrr^ | A-B/A-B | 6.50 | 5.09 | - |
|  |  | A-B/B-A | 2.50 | 1.47 | - |
| Replication terminator protein | 1f4k | A-B/A-B | - | 40.89* |  |
|  |  | A-B/B-A | - | 1.71 | - |
| ETS-1 DNA binding and autoinhibitory domain | 1k79^ | A/A | 1.20 | 4.63 | - |
|  |  | A-B/A-B | - | 1.05 | - |
|  |  | A-B/B-A | - | 22.20* | - |
| Restriction endonuclease HINCII | 1kc6^ | A-B/A-B | 3.50 | 0.94 | - |
|  |  | A-B/B-A | 4.10 | 1.38 | - |
| Transcription repressor COPG | 1ea4 | D-E/D-E | - | 39.17 | - |
|  |  | D-E/E-D | - | 48.16 | - |
|  |  | D-E/F-G | - | 53.04 | - |
|  |  | D-E/G-F | - | 40.8 | - |
| Sulfolobus solfataricus SWI2/SNF2 ATPase core domain | 1z63 | A/A | - | 50.49 | 24.47* |
| Glucocorticoid receptor | 1r4o^ | A/A | 26.00 | 44.24* | 35.09* |
|  |  | A/B | 20.20 | 25.57* | 3.43 |
| Hyperthermophile chromosomal protein SAC7D | 1azp^ | A/A | 2.30 | 2.28 | - |
| HTRF1 DNA-binding domain | 1w0t^ | A/A | 1.50 | 27.66* | - |
|  |  | A/B | - | 8.40 | - |
| Methionine repressor | 1cma | A-B/A-B | - | 23.59 | 24.80 |
|  |  | A-B/B-A | - | 19.90 | - |
| Papillomavirus type 18 E2 | 1jj4^ | A-B/A-B | 29.80 | 1.62 | - |
|  |  | A-B/B-A | 2.30 | 3.20 | - |
| T4 pyrimidine dimer specific excision repair | 1vas^ | A/A | 4.80 | 1.40 | - |
| DNA polymerase I | 4ktq^ | A/A | 1.80 | 0.75 | - |
| Organic hydroperoxide resistence transcription regulator | 1z9c^ | C-D/C-D | 5.10 | 0.18 | - |
|  |  | C-D/D-C | - | 4.24 | - |
| Diphtheria TOX repressor | 1ddn^ | A-B/A-B | 3.70 | 2.21 | - |
|  |  | A-B/B-A | - | 19.74* | - |
| Interferon Regulatory Factor 2 | 2irf^ | J/J | 2.20 | 1.97 | - |
|  |  | J/K | 23.40 | 23.06 | - |
| Multidrug binding transcription factor QACR | 1jt0^ | A-C/A-C | no decoys clustered | 31.23 | 4.09 |
|  |  | A-C/C-A | - | 31.95 | - |
| I-CreI endonuclease | 1g9z^ | A-B/A-B | 0.80 | 0.9 | - |
|  |  | A-B/B-A | 1.70 | 1.45 | - |
| Intron-encoded homing endonuclease I-PPOI | 1a73^ | A-B/A-B | 0.60 | 1.56 | - |
|  |  | A-B/B-A | 1.10 | 2.38 | - |
| Phage PHI29 transcription regulator P4 | 2fio | A-B/A-B | - | 77.13 | 31.64 |
|  |  | A-B/B-A | - | 32.26 | - |
| Adenovirus major late promotor TBP | 1qne^ | A/A | 1.40 | 0.64 | - |
| Phage lambda CII | 1zs4 | A-B-C-D/A-B-C-D | - | 13.22^#^ | 7.71 |
|  |  | A-B-C-D/D-C-B-A | - | 42.31 | - |
| ‘Difficult’ targets | | | | | |
| High mobility group protein D | 1qrv^ | A/A | 5.30 | 2.83 | - |
| CAP-CAMP | 1o3t | A-B/A-B | - | 3.69 | - |
|  |  | A-B/B-A | - | 3.30 | - |
| Epstein-Barr virus nuclear antigen-1 | 1b3t^ | A-B/A-B | 1.10 | 0.60 | - |
|  |  | A-B/B-A | 1.60 | 1.02 | - |
| Restriction endonuclease BAMHI | 3bam^ | A-B/A-B | 0.80 | 0.20 | - |
|  |  | A-B/B-A | 1.50 | 2.19 | - |
| Eco RV endonuclease | 1rva^ | A-B/A-B | 1.60 | 0.66 | - |
|  |  | A-B/B-A | 2.00 | 1.08 | - |
| Proline utilization transcription activator PUT3 | 1zme | C-D/C-D | - | 2.57 | - |
|  |  | C-D/D-C | - | 32.47* | - |
| Restriction endonuclease BGLII | 1dfm^ | A-B/A-B | 0.90 | 0.77 | - |
|  |  | A-B/B-A | 1.40 | 1.44 | - |
| Phage P22 Arc gene regulating protein | 1bdt^ | A-B/A-B | - | 42.44* | - |
|  |  | A-B/B-A | 31.50 | 38.93* | - |
|  |  | A-B/C-D | - | 4.72 | - |
|  |  | A-B/D-C | - | 54.48* | - |
| HHAI methyltransferase | 7mht | A/A | - | 0.52 | - |
| Restriction endonuclease HINP1I | 2fl3^ | A/A | 0.90 | 1.61 | - |
| PVUII endonuclease | 1eyu^ | A-B/A-B | 1.90 | 2.10 | - |
|  |  | A-B/B-A | 2.20 | 1.37 | - |
| Restriction endonuclease MVAI | 2oaa^ | B/B | 1.80 | 1.15 | - |
| **UNBOUND DOCKING** | | | | | |
| ‘Easy’ targets | | | | | |
| Phage PHI29 replication organizer protein P16.7 | 2c5r^ (2bnk/-) | A-B/A-B | - | 28.03 | 29.82 |
|  |  | A-B/B-A | - | 27.53 | - |
|  |  | A-B/C-D | - | 30.25 | 26.11 |
|  |  | A-B/D-C | 25.60 | 23.64 | - |
|  |  | A-B/E-F | 26.80 | 30.32 | 25.80 |
|  |  | A-B/F-E | 28.10 | 25.07 | - |
| Col-E7 nuclease domain | 1pt3^ (1m08/-) | A/A | 7.10 | 5.01 | - |
|  |  | A-B/A-B | - | 11.19^#^ | 5.10 |
|  |  | A-B/B-A | - | 14.10* | - |
| Sporulation specific transcription factor NDT80 | 1mnn (1mn4/-) | A/A | - | 23.48 | 22.37 |
| Restriction endonuclease FOKI | 1fok^ (2fok/-) | A/A | 2.40 | 37.01* | 3.47 |
| Papillomavirus replication initiation domain E-1 | 1ksy (1f08/-) | A-C/A-C | - | 11.74^#^ | 9.60 |
|  |  | A-C/C-A | - | 13.24* | - |
| Phage 434 CRO | 3cro (1zug/-) | L/L | - | 12.49* | 35.17* |
|  |  | L/R | - | 15.75* | 6.84 |
| Human uracil-DNA glucosylase | 1emh (1akz/-) | A/A | - | 10.64* | 12.61* |
| FADR, fatty acid responsive transcription factor | 1h9t^ (1e2x/-) | A-B/A-B | no decoys clustered | 40.37 | 8.71 |
|  |  | A-B/B-A | - | 35.82 | - |
| TRP repressor | 1tro (3wrp/-) | A-C/A-C | - | 0.65 | - |
|  |  | A-C/C-A | - | 3.23 | - |
| Retinoid X receptor DNA binding domain | 1by4^ (1rxr/-) | A/A | 30.50 | 26.01* | 30.42 |
|  |  | A/B | 28.60 | 30.21* | 22.79* |
| RUNX1 runt domain | 1hjc (1ean/-) | A/A | - | 36.66 | 14.04* |
| *E. coli* 3-methyladenine DNA glycosylase II | 1diz^ (1mpg/-) | A/A | no decoys clustered | 45.32 | 12.86* |
| Phage 434 repressor | 1rpe^ (1r63/-) | L/L | - | 8.97 | - |
|  |  | L/R | no decoys clustered | 20.22* | - |
| ‘Medium’’ targets | | | | | |
| Restriction endonuclease BSTYI | 1vrr^ (1sdo/-) | A-B/A-B | 35.30 | 25.06 | 22.98 |
|  |  | A-B/B-A | - | 25.40 | - |
| Replication terminator protein | 1f4k (1bm9/-) | A-B/A-B | - | 28.77 | 28.43 |
|  |  | A-B/B-A | - | 29.82 | - |
| ETS-1 DNA binding and autoinhibitory domain | 1k79^ (1gvj/-) | A/A | no decoys clustered | 35.85 | 19.45 |
|  |  | A-B/A-B | - | 56.51 | 21.14* |
|  |  | A-B/B-A | - | 32.50 | - |
| Restriction endonuclease HINCII | 1kc6 (2aud/-) | A-B/A-B | - | 15.07* | 21.56 |
|  |  | A-B/B-A | - | 15.93* | - |
| Transcription repressor COPG | 1ea4^ (2cpg/-) | D-E/D-E | - | 53.41 | 49.07 |
|  |  | D-E/E-D | no decoys clustered | 25.25 | - |
|  |  | D-E/F-G | - | 35.45 | 35.11 |
|  |  | D-E/G-F | - | 35.87 | - |
| Sulfolobus solfataricus SWI2/SNF2 ATPase core domain | 1z63 (1z6a/-) | A/A | - | 45.51 | 20.26 |
| Glucocorticoid receptor | 1r4o^ (1gdc/-) | A/A | 17.60 | 33.58* | 6.92 |
|  |  | A/B | 33.60 | 12.07* | 6.41 |
| Hyperthermophile chromosomal protein SAC7D | 1azp^ (1sap/-) | A/A | 8.90 | 19.67 | 7.68 |
| HTRF1 DNA-binding domain | 1w0t^ (1ba5/-) | A/A | 7.60 | 38.85 | 24.79 |
|  |  | A/B | 5.00 | 27.60 | 24.88 |
| Methionine repressor | 1cma^ (1mjk/-) | A-B/A-B | 27.80 | 20.89* | 21.17* |
|  |  | A-B/B-A | 26.50 | 23.21* | - |
| Papillomavirus type 18 E2 | 1jj4^ (1f9f/-) | A-B/A-B | 9.00 | 39.72 | 10.03^#^ |
|  |  | A-B/B-A | 10.20^#^ | 39.70 | - |
| T4 pyrimidine dimer specific excision repair | 1vas^ (1eni/-) | A/A | 9.70 | 12.12* | 10.94* |
| DNA polymerase I | 4ktq^ (1ktq/-) | A/A | 15.90 | 10.11^#^ | - |
| Organic hydroperoxide resistence transcription regulator | 1z9c (1z91/-) | C-D/C-D | - | 26.68* | 12.78* |
|  |  | C-D/D-C | - | 45.94* | - |
| Diphtheria TOX repressor | 1ddn^ (2tdx/-) | A-B/A-B | no decoys clustered | 6.47 | - |
|  |  | A-B/B-A | no decoys clustsred | 6.40 | - |
| Interferon Regulatory Factor 2 | 2irf^ (1irg/-) | J/J | 25.40 | 14.81* | 14.54* |
|  |  | J/K | - | 11.41^#^ | 11.71^#^ |
| Multidrug binding transcription factor QACR | 1jt0 (1jus/-) | A-C/A-C | - | 28.25 | 21.74* |
|  |  | A-C/C-A | - | 30.83 | - |
| I-CreI endonuclease | 1g9z^ (2o7m/-) | A-B/A-B | 19.10 | 8.85 | - |
|  |  | A-B/B-A | 19.70 | 9.38 | - |
| Intron-encoded homing endonuclease I-PPOI | 1a73^ (1evx/-) | A-B/A-B | 18.90 | 18.81 | 8.49 |
|  |  | A-B/B-A | 12.50^#^ | 22.49 | - |
| Phage PHI29 transcription regulator P4 | 2fio (2fib/-) | A-B/A-B | - | 31.23 | 23.51 |
|  |  | A-B/B-A | - | 62.79 | - |
| Adenovirus major late promotor TBP | 1qne (1vok/-) | A/A | - | 19.28^#^ | 18.21^#^ |
| Phage lambda CII | 1zs4 (1zpq/-) | A-B-C-D/A-B-C-D | - | 28.83 | 29.02 |
|  |  | A-B-C-D/D-C-B-A | - | 38.70 | - |
| ‘Difficult’ targets | | | | | |
| High mobility group protein D | 1qrv^ (1hma/-) | A/A | 9.90 | 20.96 | 10.51* |
| CAP-CAMP | 1o3t (1g6n/-) | A-B/A-B | - | 43.27 | 23.01* |
|  |  | A-B/B-A | - | 46.25 | - |
| Epstein-Barr virus nuclear antigen-1 | 1b3t^ (1vhi/-) | A-B/A-B | 12.90^#^ | 9.13 | - |
|  |  | A-B/B-A | 7.70 | 18.04* | - |
| Restriction endonuclease BAMHI | 3bam (1bam/-) | A-B/A-B | - | 19.27 | 19.86 |
|  |  | A-B/B-A | - | 20.31 | - |
| Eco RV endonuclease | 1rva (1rve/-) | A-B/A-B | - | 27.29 | 23.04 |
|  |  | A-B/B-A | - | 24.57 | - |
| Proline utilization transcription activator PUT3 | 1zme (1ajy/-) | C-D/C-D | - | 28.72 | 14.95 |
|  |  | C-D/D-C | - | 31.61 | - |
| Restriction endonuclease BGLII | 1dfm^ (1es8/-) | A-B/A-B | 34.70 | 42.66 | 29.46* |
|  |  | A-B/B-A | 36.90 | 37.72 | - |
| Phage P22 Arc gene regulating protein | 1bdt^ (1arq/-) | A-B/A-B | no decoys clustered | 40.89 | 13.93* |
|  |  | A-B/B-A | no decoys clustered | 33.88 | - |
|  |  | A-B/C-D | no decoys clustered | 53.67 | 40.58* |
|  |  | A-B/D-C | no decoys clustered | 24.18 | - |
| HHAI methyltransferase | 7mht (2hmy/-) | A/A | - | 13.35* | 17.31 |
| Restriction endonuclease HINP1I | 2fl3^ (1ynm/-) | A/A | 1.80 | 2.39 | - |
| PVUII endonuclease | 1eyu^ (1pvu/-) | A-B/A-B | 9.20 | 10.95* | 3.36 |
|  |  | A-B/B-A | 8.90 | 16.95* | - |
| Restriction endonuclease MVAI | 2oaa (2oa9/-) | B/B | - | 8.97 | - |

*** - cases with RMSD > 10 Å and with recognized protein binding site**

**^#^ - native-like decoy with RMSD > 10 Å**

**^ - complexes tested previously manually (with at least 10 near-native structures generated by GRAMM)**

**** RMSD for unbound docking – RMSD counted as a difference between measured RMSD from an unbound DNA in a decoy and minimal RMSD that can be obtained by fitting the unbound chains of DNA to the bound chains of DNA**

**Supplementary Table 2.** Results of fully automated protein-RNA docking with the NPDock server; comparison with user-supervised docking.

| Complex name | PDB code  (complex for bound and unbound cdocking, and components for unbound docking) | RMSD of ligand [Å]  unrefined  original benchmark (Tuszynska and Bujnicki, 2011) | RMSD of ligand [Å]  refined  (free docking without interface residues pair) | RMSD of ligand [Å]  refined  (docking with a constraint on one random pair of interface residues) |
| --- | --- | --- | --- | --- |
| Bound docking | | | | |
| HuD protein with (AU)-rich elements RNA | 1FXL | 2.38 | 0.49 | - |
| Nova KH domain with 20-mer RNA hairpin | 1EC6 | 3.55 | 1.57 | - |
| Poly(A)-binding protein with polyadenylate RNA | 1CVJ | 2.38 | 1.11 | - |
| U1A protein with RNA hairpin | 1URN | 0.83 | 4.91 | - |
| SRP 19 KDA protein with human SRP protein | 1JID | 29.44 | 37.49 | 36.76 |
| Unbound docking | | | | |
| Norwalk Virus polymerase with CTP/RNA primer | 3BSO  (1SH0/3BSO) | 4.18 | 8.83 | - |
| HutP/Hut mRNA | 1WPU  (1WPV/1WPU) | 2.53 | 2.35 | - |
| 9-subunit archaeal exosome/RNA | 2JEA  (2JE6/2JEA) | 12.55 | 22.9  (12.13 for fourth cluster) | 8.73 |
| SRP 19/7S.S SRP RNA | 1LNG  (1LNG/1Z43) | 7.25 | 10.96 | - |
| Synthetic Fab/P4-P6 ribozyme domain | 2R8S  (2R8S/1HR2) | 95.16 | 49.96 | 9.08 |
| SRP C-terminal domain/4.5 S RNA | 2PXV  (2PXV/1CQL) | 24.18 | 33.16 | 27.20 |
| Tyrosyl - tRNA synthetase splicing factor/group I intron RNA | 2RKJ  (1Y42/1Y0Q) | 32.12 | 79.76 | 36.38 |
| Spliceosomal 15.5 K protein/U4 snRNA fragment | 1E7K  (2JNB/1E7K) | 18.49 | 38.73 | 6.48 |

Supplementary Table 3. Results of fully automated protein-RNA docking with the NPDock server performed on the testing set proposed by Huang and Zou ([Huang and Zou, 2012](#_ENREF_1))

| Complex name | PDB code  (complex for bound and unbound cdocking, and components for unbound docking) | RMSD of ligand [Å]  refined  (free docking without interface residues pair) | RMSD of ligand [Å]  refined  (docking with a constraint on one random pair of interface residues) |
| --- | --- | --- | --- |
| **BOUND DOCKING** | | | |
| ‘Easy’ targets | | | |
| Aspartyl tRNA synthetase + Aspartyl tRNA | 1c0a | 0.54 | - |
| Ribosomal protein l25 + 5S rRNA fragment | 1dfu | 22.07 | 4.22 |
| Signal recognition particle protein + 7SL RNA | 1e8o | 53.35 | 41.49^*^ |
| 30S ribosomal protein S15 + 16S ribosomal RNA fragment | 1f7y | 2.76 | - |
| Isoleucyl-tRNA synthetase + Isoleucyl-tRNA | 1ffy | 4.91 | - |
| 30S ribosomal protein S6, S18 + 16S ribosomal RNA fragment | 1g1x | 42.0^*^ | 0.97 |
| Valyl-tRNA synthetase + tRNA(Val) | 1gax | 1.46 | - |
| Prolyl-tRNA synthetase + tRNApro(cgg) | 1h4s | 32.18 | 23.71 |
| signal recognition particle protein + 4.5S RNA domain IV | 1hq1 | 21.5 | 2.91 |
| Tyrosyl-tRNA synthetase + tRNA(Tyr) | 1j1u | 30.76^*^ | 56.99 |
| Restrictocin + Sarcin/Ricin domain RNA analog | 1jbs | 27.87 | 4.04 |
| Signal recognition particle protein + Helix 6 of human srp RNA | 1jid | 36,38 | 38.78 |
| tRNA Pseudouridine Synthase B + T Stem-Loop RNA | 1k8w | 0.78 | - |
| Threonyl-tRNA synthetase + Threonyl-tRNA synthetase mRNA | 1kog | 47.33 | 0.62 |
| Signal recognition particle protein + 7S.S srp RNA | 1lng | 0.57 | - |
| Ribosomal protein L11 + 23S ribosomal RNA fragment | 1mms | 7.33 | - |
| Glutamyl-tRNA synthetase + tRNA(Glu) | 1n78 | 0.68 | - |
| Queuine tRNA-ribosyltransferase + a stem-loop RNA substrate | 1q2r | 1.88 | - |
| Glutaminyl-tRNA synthetase + tRNA Gln II | 1qtq | 0.57 | - |
| tRNA pseudouridine synthase B + a stem-loop RNA | 1r3e | 3.01 | - |
| 30S ribosomal protein S8 + spc Operon mRNA | 1s03 | 40.68 | 3.21 |
| Small nuclear ribonucleoprotein A + Precursor form of the Hepatitis Delta virus ribozyme | 1sj3 | 2.58 | - |
| 60S ribosomal protein L30 + mRNA | 1t0k | 24.86 | 28.14 |
| 60-kDa SS-A/Ro ribonucleoprotein + Y RNA sequence, first strand, second strand | 1yvp | 2.50 | - |
| Tryptophanyl-tRNA synthetase + transfer RNA-Trp | 2ake | 30.99 | 18.48^*^ |
| Neuro-oncological ventral antigen 1 + RNA aptamer hairpins | 2anr | 44.08 | 17.14 |
| B2 protein + double-stranded RNA (dsRNA) | 2az0 | 2.13 | - |
| 23S rRNA (uracil-5-)-methyltransferase RumA + 23S ribosomal RNA fragment | 2bh2 | 0.18 | - |
| Methionyl-tRNA synthetase + tRNA(Met) | 2csx | 6.09 | - |
| SsrA-binding protein + tmRNA | 2czj | 2.72 | - |
| O-phosphoseryl-tRNA synthetase + tRNA | 2du3 | 51.79 | 53.45 |
| Ribonuclease Z + tRNA(Thr) | 2fk6 | 42.57 | 23.92^*^ |
| tRNA-splicing endonuclease + a bulge-helix-bulge RNA | 2gjw | 28.26 | 21.94^*^ |
| Coat protein + a viral RNA | 2qux | 27.51 | 28.41 |
| Probable tRNA pseudouridine synthase B + Guide RNA 1, Guide RNA 2 | 2rfk | 27.10 | 2.99 |
| a protein toxin (ToxN) + a specific RNA antitoxin (ToxI) | 2xdb | 35.67 | 35.55 |
| tRNA delta(2)-isopentenylpyrophosphate transferase + tRNA(Phe) | 2zm5 | 2.66 | - |
| Pyrrolysyl-tRNA synthetase + Bacterial tRNA | 2zni | 0.92 | - |
| Arginyl-tRNA synthetase + tRNA-Arg | 2zue | 1.23 | - |
| Toll-like receptor 3 + double-stranded RNA | 3ciy | 72.78 | 67.20 |
| Thrombin heavy chain + an RNA aptamer | 3dd2 | 35.13 | 25.45 |
| tRNA isopentenyltransferase + tRNA | 3eph | 3.67 | - |
| tRNA delta(2)-isopentenylpyrophosphate transferase + tRNA(Phe) | 3foz | 0.81 | - |
| Nucleocapsid protein +viral genomic RNA (vRNA) | 3hhz | 49.35^*^ | 69.65 |
| Probable ATP-dependent RNA helicase DDX58 + double-stranded RNA | 3lrr | 28.52 | 24.91 |
| Pseudouridine synthase Cbf5, Ribosome biogenesis protein Nop10, 50S ribosomal protein L7Ae + H/ACA RNA | 3lwr | 0.61 | - |
| ATP-dependent RNA helicase dbpA + 23S ribosomal RNA fragment | 3moj | 18.22^*^ | 5.88 |
| Polymerase + Positive-strand RNA | 3ol9 | 1.98 | - |
| CCA-Adding Enzyme + tRNA | 3ovb | 1.04 | - |
| ‘Medium’ targets | | | |
| Arginyl-tRNA synthetase + tRNA(Arg) | 1f7u | 0.24 | - |
| Aspartyl-tRNA synthetase+Aspartyl transfer RNA | 1il2 | 49.37 | 1.73 |
| Core protein P19 + small interfering RNA | 1r9f | 37.66 | 16.92^*^ |
| Ribonuclease III + double-stranded RNA (dsRNA) | 1rc7 | 7.94 | - |
| Seryl-tRNA synthetase + tRNAser | 1ser | 71.73 | 31.87^*^ |
| Transcription factor IIIA +5S ribosomal RNA fragment | 1un6 | 38.50^*^ | 43.73 |
| Aminoacyl-tRNA synthetase + tRNA(Leu) transcript with anticodon cag | 2bte | 64.01 | 41.90 |
| Methionyl-tRNA fMet formyltransferase + Formyl-methionyl-tRNAfMet2 | 2fmt | 41.10 | 7.23 |
| Ribonuclease III + double-stranded RNA | 2nug | 3.82 | - |
| Selenocysteine-specific elongation factor + SECIS mRNA | 2uwm | 39.02 | 29.83 |
| 50S ribosomal protein L1 + Fragment of mRNA for L1-operon containing regulator L1-binding site | 2vpl | 1.48 | - |
| Non-structural protein 1 + double-stranded RNA (dsRNA) | 2zko | 2.64 | - |
| Uncharacterized protein MJ0883 + tRNA(Leu) | 2zzm | 49.33 | 22.29^*^ |
| L-seryl-tRNA(Sec) kinase + Selenocysteine tRNA | 3add | 46.65 | 24.32 |
| Dimethyladenosine transferase + 16S rRNA fragment | 3ftf | 23.42^*^ | 35.92 |
| O-phosphoseryl-tRNA(Sec) selenium transferase + tRNASec | 3hl2 | 66.59 | 22.82^*^ |
| ‘Difficult’ targets | | | |
| tyrosyl-tRNA synthetase + Wild-type tRNAtyr(Gua) | 1h3e | 30.89 | 31.30 |
| Nuclear factor NF-kappa-B p105 subunit + RNA aptamer | 1ooa | 28.47 | 5.14 |
| Cysteinyl-tRNA synthetase + Cysteinyl tRNA | 1u0b | 0.82 | - |
| 50S ribosomal protein L1 + mRNA | 2hw8 | 0.53 | - |
| Iron-responsive element-binding protein 1 + Ferritin IRE RNA | 2ipy | 22.67 | 19.87^*^ |
| Fab heavy chain, Fab light chain + p4-p6 RNA ribozyme domain | 2r8s | 37.33 | 13.56^#^ |
| Signal recognition 54 kDa protein + 7S.S SRP RNA | 2v3c | 7.76 | - |
| **UNBOUND DOCKING** | | | |
| ‘Easy’ targets | | | |
| Aspartyl tRNA synthetase + Aspartyl tRNA | 1c0a  (1il2/1efw) | 64.32 | 5.19 |
| Ribosomal protein l25 + 5S rRNA fragment | 1dfu  (3ofq/1feu) | 2.82 | - |
| Signal recognition particle protein + 7SL RNA | 1e8o  (1e8o/1ry1) | 34.36 | 59.59 |
| 30S ribosomal protein S15 + 16S ribosomal RNA fragment | 1f7y  (2vqe/1dk1) | 1.76 | - |
| Isoleucyl-tRNA synthetase + Isoleucyl-tRNA | 1ffy  (1qu3/1qu2) | 3.62 | - |
| 30S ribosomal protein S6, S18 + 16S ribosomal RNA fragment | 1g1x  (2vqe/1g1x) | 39.81 | 1.47 |
| Valyl-tRNA synthetase + tRNA(Val) | 1gax  (1gax/1ivs) | 1.60 | - |
| Prolyl-tRNA synthetase + tRNApro(cgg) | 1h4s  (1hc7/1h4q) | 62.68 | 9.24 |
| signal recognition particle protein + 4.5S RNA domain IV | 1hq1  (3lqx/1dul) | 23.26 | 5.11 |
| Tyrosyl-tRNA synthetase + tRNA(Tyr) | 1j1u  (1u7d/-) | 29.84 | 23.17^*^ |
| Restrictocin + Sarcin/Ricin domain RNA analog | 1jbs  (1jbr/1jbt) | 40.66 | 6.27 |
| Signal recognition particle protein + Helix 6 of human srp RNA | 1jid  (3ktv/1l1w) | 36.11 | 42.17 |
| tRNA Pseudouridine Synthase B + T Stem-Loop RNA | 1k8w  (1r3f/1zl3) | 22.04 | 18.54^*^ |
| Threonyl-tRNA synthetase + Threonyl-tRNA synthetase mRNA | 1kog  (1evl/1kog) | 30.84^*^ | 23.02 |
| Signal recognition particle protein + 7S.S srp RNA | 1lng  (3ndb/2v3c) | 1.81 | - |
| Ribosomal protein L11 + 23S ribosomal RNA fragment | 1mms  (2jq7/1oln) | 4.14 | - |
| Glutamyl-tRNA synthetase + tRNA(Glu) | 1n78  (1j09/2dxi) | 5.97 | - |
| Queuine tRNA-ribosyltransferase + a stem-loop RNA substrate | 1q2r  (1r5y/1q2s) | 38.77 | 2.82 |
| Glutaminyl-tRNA synthetase + tRNA Gln II | 1qtq  (1gtr/1qrs) | 1.63 | - |
| tRNA pseudouridine synthase B + a stem-loop RNA | 1r3e  (1ze2/-) | 1.10 | - |
| 30S ribosomal protein S8 + spc Operon mRNA | 1s03  (3ofo/1so3) | 30.02 | 4.19 |
| Small nuclear ribonucleoprotein A + Precursor form of the Hepatitis Delta virus ribozyme | 1sj3  (1m5o/1vc7) | 5.34 | - |
| 60S ribosomal protein L30 + mRNA | 1t0k  (3o58/-) | 21.19 | 22.13 |
| 60-kDa SS-A/Ro ribonucleoprotein + Y RNA sequence, first strand, second strand | 1yvp  (1yvr/1yvp) | 31.97 | 19.48 |
| Tryptophanyl-tRNA synthetase + transfer RNA-Trp | 2ake  (2dr2/2azx) | 36.13 | 40.56 |
| Neuro-oncological ventral antigen 1 + RNA aptamer hairpins | 2anr  (-/2ann) | 46.52 | 25.51 |
| B2 protein + double-stranded RNA (dsRNA) | 2az0  (2b9z/2az2) | 12.0^#^ | 6.95 |
| 23S rRNA (uracil-5-)-methyltransferase RumA + 23S ribosomal RNA fragment | 2bh2  (1uwv/2bh2) | 2.21 | - |
| Methionyl-tRNA synthetase + tRNA(Met) | 2csx  (2csx/2ct8) | 24.50 | 6.32 |
| SsrA-binding protein + tmRNA | 2czj  (1wjx/2czj) | 12.30 | 22.55 |
| O-phosphoseryl-tRNA synthetase + tRNA | 2du3  (2du5/2du4) | 50.42 | 53.19 |
| Ribonuclease Z + tRNA(Thr) | 2fk6  (1y44/-) | 40.72 | 24.58 |
| tRNA-splicing endonuclease + a bulge-helix-bulge RNA | 2gjw  (1r0v/-) | 16.90 | 25.66 |
| Coat protein + a viral RNA | 2qux  (2qud/2qux) | 29.75 | 23.37 |
| Probable tRNA pseudouridine synthase B + Guide RNA 1, Guide RNA 2 | 2rfk  (3lwr/3hjy) | 4.93 | - |
| a protein toxin (ToxN) + a specific RNA antitoxin (ToxI) | 2xdb  (2xd0/-) | 3.47 | - |
| tRNA delta(2)-isopentenylpyrophosphate transferase + tRNA(Phe) | 2zm5  (3foz/2zxu) | 0.53 | - |
| Pyrrolysyl-tRNA synthetase + Bacterial tRNA | 2zni  (2znj/2zni) | 50.90 | 39.69 |
| Arginyl-tRNA synthetase + tRNA-Arg | 2zue  (-/2zuf) | 1.07 | - |
| Toll-like receptor 3 + double-stranded RNA | 3ciy  (3cig/-) | 42.38^*^ | 36.81 |
| Thrombin heavy chain + an RNA aptamer | 3dd2  (1gj5/-) | 34.42 | 28.80 |
| tRNA isopentenyltransferase + tRNA | 3eph  (3epk/3epj) | 1.24 | - |
| tRNA delta(2)-isopentenylpyrophosphate transferase + tRNA(Phe) | 3foz  (2zxu/2zm5) | 2.05 | - |
| Nucleocapsid protein +viral genomic RNA (vRNA) | 3hhz  (3ptx/2gic) | 67.98^*^ | 48.22 |
| Probable ATP-dependent RNA helicase DDX58 + double-stranded RNA | 3lrr  (3lrn/-) | 36.57 | 39.97 |
| Pseudouridine synthase Cbf5, Ribosome biogenesis protein Nop10, 50S ribosomal protein L7Ae + H/ACA RNA | 3lwr  (3lwp/3hjw) | 1.15 | - |
| ATP-dependent RNA helicase dbpA + 23S ribosomal RNA fragment | 3moj  (2goc/-) | 18.30 | 15.64^*^ |
| Polymerase + Positive-strand RNA | 3ol9  (3ol6/3olb) | 1.21 | - |
| CCA-Adding Enzyme + tRNA | 3ovb  (3ov7/3ouy) | 33.64 | 0.87 |
| ‘Medium’ targets | | | |
| Arginyl-tRNA synthetase + tRNA(Arg) | 1f7u  (1bs2/1f7v) | 46.67 | 20.87 |
| Aspartyl-tRNA synthetase+Aspartyl transfer RNA | 1il2  (1eqr/1asy) | 48.85 | 16.61^#^ |
| Core protein P19 + small interfering RNA | 1r9f  (-/3cz3) | 42.13^*^ | 18.63 |
| Ribonuclease III + double-stranded RNA (dsRNA) | 1rc7  (1yyo/1di2) | 6.50 | - |
| Seryl-tRNA synthetase + tRNAser | 1ser  (1ses/-) | 54.94 | NO |
| Transcription factor IIIA +5S ribosomal RNA fragment | 1un6  (2hgh/1un6) | 37.71 | 15.64 |
| Aminoacyl-tRNA synthetase + tRNA(Leu) transcript with anticodon cag | 2bte  (1h3n/2byt) | 24.87^*^ | NO |
| Methionyl-tRNA fMet formyltransferase + Formyl-methionyl-tRNAfMet2 | 2fmt  (1fmt/3cw5) | 2.24 | - |
| Ribonuclease III + double-stranded RNA | 2nug  (2nuf/-) | 14.46 | 3.06 |
| Selenocysteine-specific elongation factor + SECIS mRNA | 2uwm  (1lva/1wsu) | 39.37 | 23.03 |
| 50S ribosomal protein L1 + Fragment of mRNA for L1-operon containing regulator L1-binding site | 2vpl  (2ov7/1u63) | 25.66 | 36.18 |
| Non-structural protein 1 + double-stranded RNA (dsRNA) | 2zko  (2z0a/2zi0) | 42.43 | 31.70 |
| Uncharacterized protein MJ0883 + tRNA(Leu) | 2zzm  (2zzn/-) | 47.84 | 29.82 |
| L-seryl-tRNA(Sec) kinase + Selenocysteine tRNA | 3add  (3adc/2adb) | 46.85 | 56.55 |
| Dimethyladenosine transferase + 16S rRNA fragment | 3ftf  (3ftd/3fte) | 23.03^*^ | 23.02^*^ |
| O-phosphoseryl-tRNA(Sec) selenium transferase + tRNASec | 3hl2  (3hl2/3a3a) | 42.99 | 27.73 |
| ‘Difficult’ targets | | | |
| tyrosyl-tRNA synthetase + Wild-type tRNAtyr(Gua) | 1h3e  (1h3f/-) | 33.15 | 50.99 |
| Nuclear factor NF-kappa-B p105 subunit + RNA aptamer | 1ooa  (1nfk/2jwv) | 40.94 | 25.93 |
| Cysteinyl-tRNA synthetase + Cysteinyl tRNA | 1u0b  (1li5/1b23) | 27.64 | 14.63^#^ |
| 50S ribosomal protein L1 + mRNA | 2hw8  (1ad2/1zho) | 29.55 | 22.88 |
| Iron-responsive element-binding protein 1 + Ferritin IRE RNA | 2ipy  (2b3y/2ipy) | 31.75 | 31.71 |
| Fab heavy chain, Fab light chain + p4-p6 RNA ribozyme domain | 2r8s  (3ivk/1hr2) | 63.81 | 81.24 |
| Signal recognition 54 kDa protein + 7S.S SRP RNA | 2v3c  (3ndb/1lng) | 38.42 | 17.99^*^ |

NO - there was no decoys that fulfill the restrains

* - cases with RMSD > 10 Å and with recognized protein binding site

^#^ - native-like decoy with RMSD > 10 Å

**REFERENCES**

Huang, S.Y. and Zou, X. (2012) A nonredundant structure dataset for benchmarking protein-RNA computational docking, *J Comput Chem*.

Munteanu, M.G.*, et al.* (1998) Rod models of DNA: sequence-dependent anisotropic elastic modelling of local bending phenomena, *Trends Biochem Sci*, **23**, 341-347.

Tuszynska, I. and Bujnicki, J.M. (2011) DARS-RNP and QUASI-RNP: New statistical potentials for protein-RNA docking, *BMC Bioinformatics*, **12**, 348.

van Dijk, M. and Bonvin, A.M. (2008) A protein-DNA docking benchmark, *Nucleic Acids Res*, **36**, e88.
